# How elevated nitrogen load affects bacterial community structure and nitrogen cycling services in coastal water

**DOI:** 10.3389/fmicb.2022.1062029

**Published:** 2022-12-22

**Authors:** Linus Shing Him Lo, Zhimeng Xu, Sangwook Scott Lee, Wing Keung Lau, Jian-Wen Qiu, Hongbin Liu, Pei-Yuan Qian, Jinping Cheng

**Affiliations:** ^1^Department of Ocean Science, The Hong Kong University of Science and Technology, Kowloon, Hong Kong SAR, China; ^2^The Southern Marine Science and Engineering Guangdong Laboratory (Guangzhou), Guangzhou, China; ^3^Department of Biology, Hong Kong Baptist University, Kowloon, Hong Kong SAR, China; ^4^Department of Science and Environmental Studies, The Education University of Hong Kong, Tai Po, Hong Kong SAR, China

**Keywords:** microbial community, coastal aquaculture, nitrogen cycle, ecological functions, coastal water

## Abstract

Nutrient pollution in the coastal environment has been accelerated by progressively intensifying aquaculture activities. Excessive nutrients can lead to coastal eutrophication with serious economic and ecological consequences. In this study, we studied coastal planktonic microbial community over a year to understand the aquaculture impact on coastal water quality and function. We observed increased total inorganic nitrogen concentrations in active fish farms to favor the diverse Alpha- and Gammaproteobacteria. Bacterial community alpha diversity in fish farms was positively correlated with total inorganic nitrogen, and active fish farming co-influenced the bacterial structural composition and regional beta diversity. By analyzing the nitrogen cycle-related functional compositions and pathways using PICRUSt2 prediction on inferred genomes, we identified the contribution of over 600 bacterial species to four major pathways. Enhanced nitrogen load in active fish farms was positively correlated with elevated dissimilatory nitrate reduction and denitrification pathway abundances. Fallowed fish farms were characterized by a predicted high abundance of *nirA* and *narB* genes contributing to assimilatory nitrate reduction pathway due to the prevalence of Cyanobacteria. Overall, these results suggested active operation and short hiatus in coastal aquaculture practices could rapidly impact planktonic bacterial communities and further influence nitrogen cycling and associated processes. These findings will improve the understanding of the responses and interactions between microbiome and aquaculture activities. In a world of increasing aquaculture demands, this work has important implications for sustainable water resource management and development.

## Introduction

The important aquatic ecosystems and their associated biodiversity and ecological functioning has been inevitably impacted by human activities. This resulted in elevated global awareness and increasing demands for sustainable use of aquatic resources. Aquaculture is one of the forefronts in utilizing the rich biological resources in aquatic ecosystems. Aquaculture is an important food source and contributed over 80 million metric tons of global fish production (FAO, 2020). This production is expected to rise with increasing population demands, putting incentive into higher intensity coastal aquaculture for both the leading producers in mainly South and Southeast Asian countries, as well as the developing coastal countries with often inadequate scientific expertise and supporting infrastructure.

One of the major concerns of high intensity coastal aquaculture lies in its high nutrient loading, causing potential nutrient pollution and water quality deterioration in its immediate and adjacent environments. In 2018, global aquafeed output was estimated to surpass 40 million metric tons (Kong et al., 2020). Fish feeds that are not fully consumed alongside fish excreta are significant sources of dissolved inorganic nutrients in water. In Chinese coastal seas, it is estimated that only 25 and 13% of total nitrogen and phosphorus in feed for mariculture were retained in harvest, causing aquaculture production to be a spatially concentrated source of nutrient in Chinese waters (Wang et al., 2019). Fish farm nutrient dispersal can contribute to coastal eutrophication and is detrimental to the environment. In light of intensifying aquaculture production, there is preliminary evidence that higher production fish farms with presumably higher loading to have longer nutrient dispersal ranges, detected in distances up to 2 km from the source (García-Sanz et al., 2011). While nutrients in open sites may have their concentrations more rapidly diluted, semi-enclosed bays could be more vulnerable to eutrophication (Lee et al., 2018; Altieri and Diaz, 2019). Subsequently, an elevated nutrient level in the surrounding waters of fish farms is favorable for harmful algal blooms, which are capable of causing mass fish kills and reportedly loss in tourism revenue up to USD 500 million evidenced in the Black Sea (Rabotyagov et al., 2020). In many cases, dedicated aquaculture zones are not distant to waters with public access such as piers or beaches. Thus, aquaculture practice and input, nutrient loading, and biogeochemical cycles involved are among the fundamental processes that require careful monitoring and management to prevent environmental and human health consequences arising from polluted waters. This in turn can allow better utilization of coastal water resources and achieve sustainable development.

Microorganisms are a key component in biogeochemical cycles across ecosystems as they contribute to decomposition processes and recycling of essential nutrients, which in turn the biomass production supports the higher trophic levels of the food web (Kieft et al., 2018). In particular, nitrogen is an important primary building block of biomass that is highly demanded in aquatic ecosystems and supplied in fish feed (Streicher et al., 2021). Fluctuations in nitrogen load is responded by microorganisms in the aquatic environment as they are responsible for the various nitrogen transforming reactions associated with the nitrogen cycle. The general view of microbial transformations in nitrogen cycle often involves microorganisms classified to processes, such as “nitrifiers,” “denitrifiers,” “N_2_-fixers” responsible for nitrification, denitrification, nitrogen fixation, and more (Kuypers et al., 2018). However, there is increasing evidence for metabolic versatility in nitrogen-transforming microorganisms where organisms can perform multiple processes such as fixing nitrogen gas and denitrify simultaneously (Hutchins and Capone, 2022). This brings forth the need to better examine nitrogen cycle-related processes with considerations of the diverse contributors beyond classic classifications. It is well established that environmental gradients can correlate well with compositional shifts in microbial communities (Mai et al., 2018; Li et al., 2021). However, the extent to which emerging high intensity aquaculture may impact microbial communities and associated nitrogen cycling services contributed has yet to be clearly understood, particularly for the coastal marine ecosystems.

Coastal aquaculture zones are simultaneously naturally dynamic and human-manipulated, where a multitude of influential factors co-exist and co-impact microbial communities. The increased nitrogen load from high intensity aquaculture and agriculture (Wang et al., 2018) have resulted in a global increase in coastal eutrophication. Coastal eutrophication can lead to hypoxia and anoxia, habitat degradation, reduction in biodiversity, altered food-webs, and increase of harmful algal blooms (Howarth, 2008). Thus, the aquacultural introduction of nitrogen nutrients and their potential knock-on impact on microbial communities and associated nutrient cycling processes in fish farm waters are significant knowledge gaps that require further investigations. Understanding such influence is important to prevent undesired outcomes and tackle the problem at its core, but to best of our knowledge, relevant studies have been limited in incorporating microbial ecology components. Concentrated nutrient input can represent potential dispersal points of nutrient, waste, as well as specific bacterial community from fish farm waters to surrounding environment, in which a greater biogeographical scale impact on environmental and human health than intended cannot be dismissed.

In this study, we used coastal water zones of Hong Kong as a model to investigate bacterial community structural and nitrogen cycle-related functional changes under fish farm activity influence over a year long period. We applied an environmental DNA approach to investigate aquaculture impact on free-living microbial communities. We hypothesized that structural composition and nitrogen cycle-related functional contribution by bacteria were dynamically affected by aquaculture practices with continuous nutrient input. The aims of this study were to (1) characterize the spatiotemporal variation and aquacultural impact on total bacteria compositions in coastal aquaculture waters, and (2) investigate the impact on nitrogen cycle-related functional taxa groups imposed by aquaculture activity in Hong Kong coastal waters. This study will serve as an important step to achieve a better understanding of microbial communities and nitrogen cycle-related functional potentials in an aquaculture setting. The findings of the study will not only form a beneficial reference for monitoring coastal bacterial communities, but also have direct implications for the management of coastal waters and associated human safety concerns.

## Materials and methods

### Site description and sample collection

**Figure 1:**
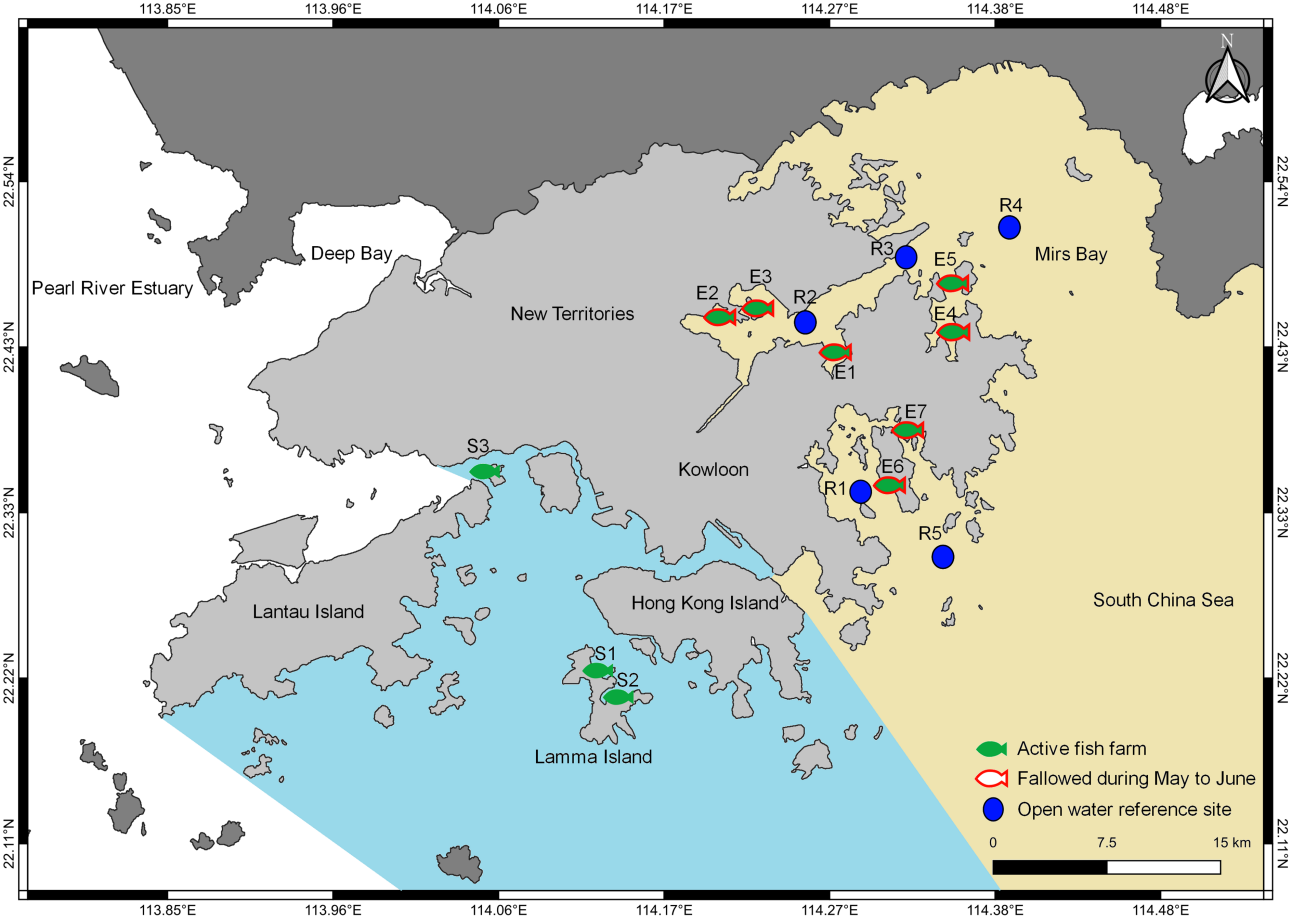
Map of sampling sites in Hong Kong conducted between September 2020 and August 2021. Fish farm sites were indicated based on temporal aquaculture activity and grouped according to geographic location.

Hong Kong is a fast-developing coastal city located at the south of China and represents a subtropical to temperate climate zone where coastal waters are of various levels of impacted quality subjected to local water quality regulations. In this study, surface seawater samples were collected from a combined total of 15 eastern and southern sites of Hong Kong, as shown in Figure 1. Monthly to bimonthly sampling, depending on access availability, was conducted for a total of 11 coastal sites between September 2020 to August 2021, which consisted of ten local fish farms (FF) within dedicated fish culture zones (FCZs), and one reference site (RS). The fish farm sampling sites were E1–E7 (*n* = 49) and S1–S3 (*n* = 24) from eastern and southern waters, respectively, and the reference site was R1 (*n* = 8) with no fish farming activities. An additional four open water reference sites R2 to R5 with no fish farming activities and historically similar background environment and nutrient conditions according to the Environmental Protection Department (EPD) of Hong Kong^1^ were sampled during June 2021 (*n* = 4) and compiled in this study; they serve as month-wise replicates to reinforce a study focus on the eastern region during the wet season. A total of 85 samples was collected and used in this study. Detailed sample information can be found in Supplementary Table 1.

At each sampling site, 1 l of surface seawater (1 m depth) was sampled and stored in a sterile bottle in an icebox, before transporting back to the laboratory for filtering. A vacuum pump was used to sequentially filter samples through four 0.22 μm pore size polycarbonate membranes (Millipore Corporation, United States) in 4 aliquots of 250 ml. All filter units were labelled and stored at −80°C until DNA extraction. All samples were transported and handled within 6 h of collection on the day to minimize possible sample degradation.

### Environmental parameter measurement

For each fish farming site, hydrological parameters of seawater, including temperature, dissolved oxygen (DO), and pH, were measured *in situ* during the sampling process using the EXO2 multiparameter water quality sonde (YSI, United States). Chlorophyll-a level was measured using Trilogy Laboratory Fluorometer (Turner Designs, United States). Total inorganic nitrogen (TIN) was measured using a San++ Continuous Flow Analyzer (Skalar, Netherlands). Other environmental parameters from corresponding monitoring stations in the proximity of each sampling site, set up by the EPD (see Footnote 1), were previously collected and compiled to inform and supplement site selection and the study. These include 5-day biochemical oxygen demand, ammonia, chlorophyll-a, dissolved oxygen, *E. coli*, faecal coliform, nitrate, nitrite, pH, salinity, secchi disc depth, suspended solids, temperature, total inorganic nitrogen, total nitrogen, total phosphorus and turbidity.

### Environmental DNA extraction

The environmental DNA was collected on the filter membranes from seawater samples.

Total DNA extraction was carried out following a modified SDS-based method described by Zhang et al. (2008). In brief, stored filter membranes were subjected to freeze and thaw three times at −80°C and at 65°C to facilitate cell lysis process. Proteinase K and sodium dodecyl sulphate (SDS) were added into genomic digestion buffer and incubated for 1 h at 65°C. The buffer was then mixed with an equal volume of phenol:chloroform:isoamyl alcohol (25:24:1), vortexed and centrifuged at 12000 rpm for 10 min. The supernatant was carefully collected into new tubes and the extraction process was repeated two times. Isopropyl alcohol of 70% of the volume of the solution was then added and vortexed before storing at −20°C overnight. Solutions were then centrifuged at 13300 rpm for 15 min and supernatants were decanted. Finally, 70% ethanol was added for washing and extracted purified DNA was resuspended in 50 μl of TE buffer and stored at −20°C until further process. Quality and concentration of extracted DNA from seawater samples were verified using a BioDrop spectrophotometer (Biochrom, United Kingdom) and calculated before subsequent analysis.

### PCR amplification and 16S rRNA sequencing

The V3 - V4 variable region of the 16S rRNA gene was targeted using the 341F (5′ -CCTAYGGGRBGCASCAG - 3′) and 806R (5′ - GGACTACNNGGGTATCTAAT - 3′) primer pair for PCR amplification (Herlemann et al., 2011). In brief, a total reaction volume of 20 μl PCR mixture containing 2 μl DNA template, 6 μl ddH_2_O, 10 μl 2 × Taq PCR Mastermix (TIANGEN, China), and 2 μl forward and reverse primers were conducted on a thermocycler. The amplification process was as follows: an initial activation step at 94°C for 1 min, followed by 35 cycles of 30 s denaturation at 95°C, annealing at 56°C for 30 s, extension at 72°C for 30 s, and a final elongation step at 72°C for 10 min. The PCR products were verified by 2% agarose gel electrophoresis. Before sequencing, the PCR products were pooled into an equimolar concentration of 10 nM/sample to produce a similar sequencing depth per sample. Sequencing was performed by Novogene (Beijing, China) on the Illumina NovaSeq platform (Illumina, USA) using paired-end sequencing. The sequence reads generated are deposited in the National Center for Biotechnology Information (NCBI) Sequence Read Archive under the accession number (PRJNA908141).

### Sequencing data processing

The sequencing data were processed and analyzed using QIIME2 (version 2021.11; Bolyen et al., 2019). First, the sequencing data were demultiplexed according to barcode sequences. Paired-end fastq.gz files and a manifest metadata file were imported to build the QIIME2 artifacts. Deblur (Amir et al., 2017) pipeline plugin in QIIME2 was used to control the quality of sequences and to construct a feature table containing the distribution of ASVs (amplicon sequence variants). The Deblur pipeline involves quality filtering, dereplication, and chimera identification. The ASVs were taxonomically assigned according to the SILVA database (release 138; Quast et al., 2012) using the feature-classifier QIIME2 plugin. The ASVs which cannot be classified beyond the bacteria domain level or were classified as chloroplast and mitochondria were removed from downstream analysis. Alpha and beta diversity of bacterial communities were calculated using QIIME2 core-metrics plugin. The alpha diversity of bacterial communities was determined based on observed unique features, Shannon index, and Pielou’s evenness. Rarefaction curves for random sampling were established to confirm adequate sequencing depth before inclusion. A phylogenetic tree based on sequence alignments was subsequently constructed using FastTree (Price et al., 2010) with Multiple Alignment using Fast Fourier Transform (MAFFT; Katoh et al., 2002) tool.

PICRUSt2 was used to functionally predict and annotate the microbial community composition based on 16S rRNA marker gene and the Kyoto Encyclopedia of Genes and Genomes (KEGG) database (Douglas et al., 2020). A stratified table of KEGG orthologs (KOs) was produced. The predicted KO relative abundance was calculated and grouped into KEGG metabolic pathways. Bacteria of which the KOs were annotated to were considered to contribute to the respective KEGG pathways. The nearest sequenced taxon index (NSTI) was calculated to quantify the availability of the nearest sequenced reference genome, which was used to verify whether PICRUSt predictions were reasonable (Langille et al., 2013).

### Statistical analysis

The normality of data was tested prior analysis using Shapiro–Wilk normality test. The differences in alpha diversity between bacterial communities were tested using Mann–Whitney U test. The analysis of beta diversity was conducted through a Principal Coordinate Analysis (PCoA) based on Bray–Curtis dissimilarity matrix, using the QIIME2 pipeline. Differences between microbial communities were analyzed through Permutational Multivariate Analysis of Variance (PERMANOVA, Adonis test) using QIIME2 (Anderson, 2014). A dendrogram was then constructed based on Ward’s hierarchical clustering to visualize the relationship between bacterial communities.

Analysis of the composition of microbiomes (ANCOM; Mandal et al., 2015) based on the W-statistic was performed using QIIME2 to determine differentially abundant taxa between sample sites. The use of ANCOM to calculate pairwise log-ratios between combinations of taxa and determine the number of times (W) the null hypothesis of “no difference between each pairwise comparison of taxa” can be rejected was performed as previously demonstrated (Zhang L. et al., 2022).

Differences in physicochemical properties and concentrations were analyzed using independent-samples *t*-test. Normality of data was tested with Shapiro–Wilk normality test. Spearman’s correlation was used to investigate the relationship between environmental parameters, bacterial community taxa and gene abundances, and diversity indices. Unless otherwise specified, all statistical analyses and visualization were conducted using R Core Team (2019) and Prism 9 (GraphPad, United States) software. Phylogenetic tree visualization was conducted using the Interactive Tree of Life (iTOL; Letunic and Bork, 2021) tool. Statistical differences were considered significant when *p* < 0.05.

## Results

### Increased inorganic nitrogen and eDNA concentration in active fish farming waters

**Figure 2:**
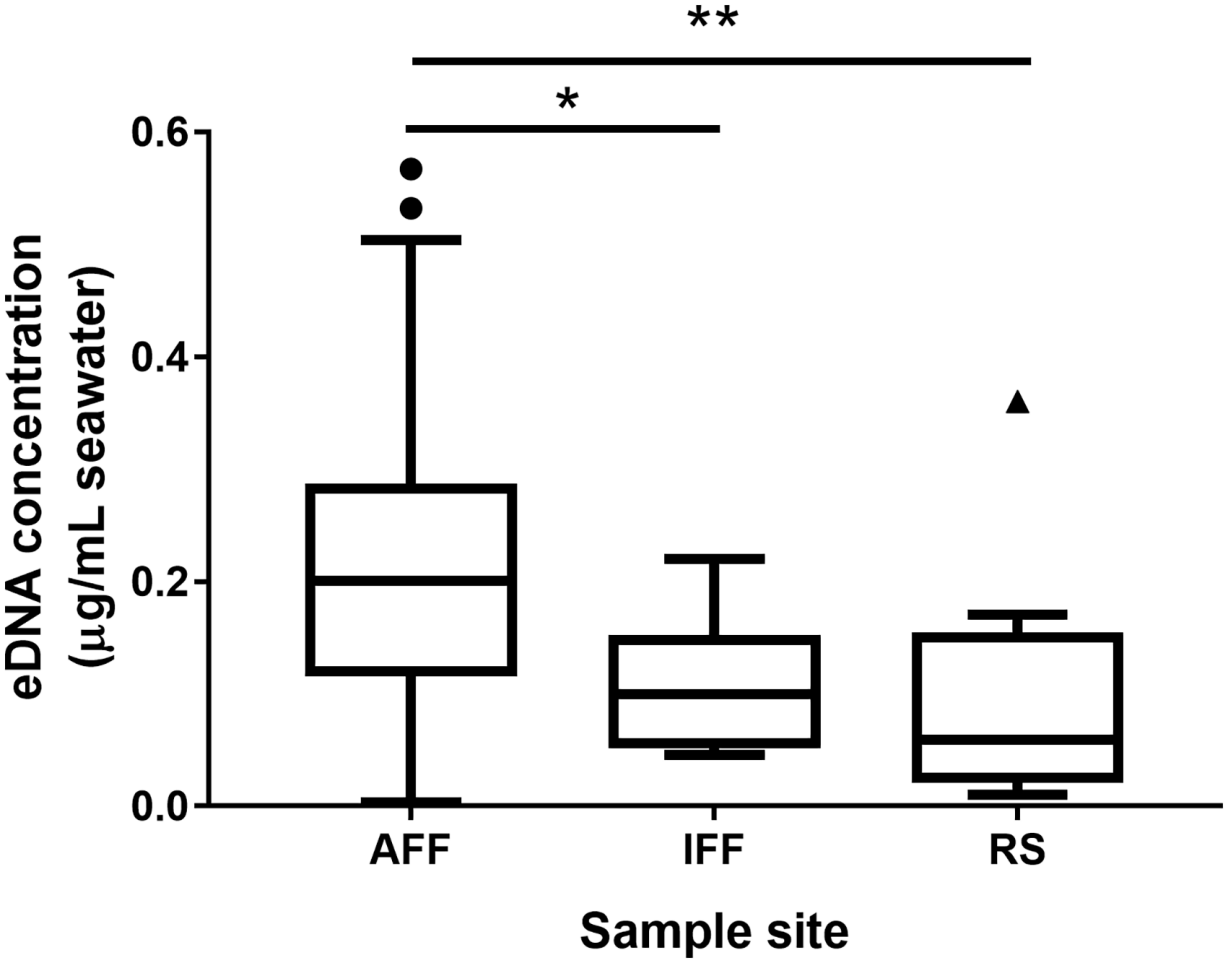
Environmental DNA concentrations in sampled Hong Kong fish farms and open waters. Asterisks indicate statistically significant difference (Kruskal-Wallis test, *p* < 0.05). AFF, active fish farms; IFF, inactive fish farms; RS, open water reference sites. Asterisks indicate statistically significant difference according to corresponding statistical tests mentioned in figure i.e., Mann Whitney U-test or Kruskal-Wallis test. * indicates *p*  < 0.05 and ** indicates *p*  < 0.01.

The measured chemical parameters in fish farm water samples, including pH, TIN, and chlorophyll-a, were summarized in Table 1 (see Supplementary Table 1 for detailed information). Fish farm samples were distinguished into active or inactive fish farm samples (hereby AFF and IFF) by TIN concentration measurements obtained. Here, fish farm samples with a TIN concentration that was near or lower than the minimum background TIN concentration in the nearest local EPD monitoring station in open waters (10 μg/l; Environmental Protection Department, 2020), were considered as having minimal nutrient input for feeding purposes. IFF was mainly observed during May and June in eastern FF sites. A peak in TIN concentration was recorded during September in site S3 at 441 μg/l, which is approaching an order higher than the annual average for eastern sites such as E1 (58 μg/l). Recorded physical parameters such as temperature between fish farm water samples and reference site samples generally do not differ within the same month (*p* < 0.05; Supplementary Table 1). For chemical parameters, pH and chlorophyll-a values recorded between fish farm sites do not statistically differ. The normalized total environmental DNA concentration in sampled water was summarized in Figure 2. Results of pairwise Kruskal-Wallis test showed that seawater in AFF sites on average contained more genetic material than IFF and RS, attributed to the higher biomass of the site.

**Table 1:** Seawater parameters between active and inactive periods of fish farms.

Sample sites	Active fish farm samples			Inactive fish farm samples			pH	TIN (μg/L)	Chl-a (μg/L)	pH	TIN (μg/L)	Chl-a (μg/L)
E1	8.045	66.885	5.343	7.960	3.000	1.530
E2	8.121	79.780	6.323	8.050	5.840	1.460
E3	7.948	72.135	4.158	7.950	3.000	2.440
E4	8.067	55.665	3.080	8.000	10.780	1.010
E5	8.071	66.784	2.203	7.960	7.610	0.450
E6	8.030	46.350	1.792	7.930	3.000	0.990
E7	8.184	65.753	3.075	8.020	3.000	7.740

TIN, Total Inorganic Nitrogen; Chl-a, Chlorophyll-a.

### Active fish farming activities select for Proteobacteria subtaxa and pathogenic bacterial groups

**Figure 3:**
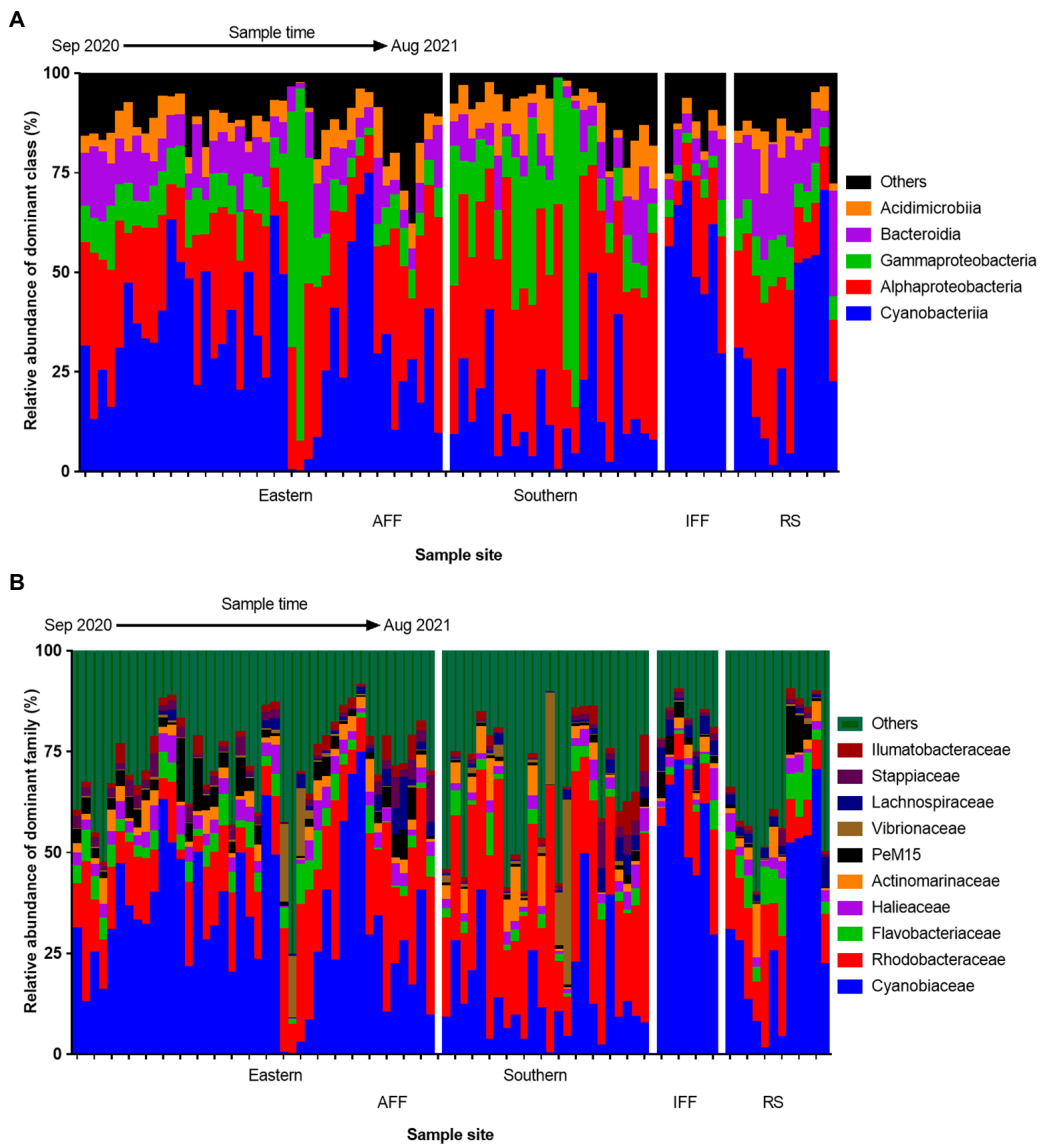
Spatiotemporal variation in bacterial community compositions at **(A)** class and **(B)** family levels among Hong Kong coastal waters. All samples were grouped based on their geographic location in Hong Kong and sub-ordered by sampling time from September 2020 to August 2021. AFF, active fish farms; IFF, inactive fish farms; RS, open water reference sites.

The spatiotemporal variation in composition of total bacterial community in Hong Kong coastal waters was summarized at the class and family level to visualize dominant bacterial group structures (Figure 3). At the class level (Figure 3A), Cyanobacteria and Alphaproteobacteria were the dominant bacterial groups that inhabits coastal waters, recorded with a mean relative abundance of 29.3 and 29.0% over the year, respectively. In IFFs, the relative abundance of Cyanobacteria appeared to be uniformly higher compared to those in AFFs (IFF: 54.0%, AFF: 26.2%, on average), favoring the growth of autotrophic producers. In contrast, both the classes Alpha- and Gammaproteobacteria had generally higher relative abundance in AFF compared to IFF sites. These two classes are known to be highly diverse bacterial contributors to ecological functions, while Gammaproteobacteria is also known to harbor many potential taxa of attention in terms of human and aquaculture pathogens. Bacteroidia was the only major class observed to have a uniform high relative abundance in RS compared to FF sites (RS: 17.6%, IFF: 7.4%, AFF: 8.8%, on average), with also a potential spatial variation between eastern and southern sites.

At the family level, the composition of the top ten dominant family with mean relative abundance >1% is shown in Figure 3B. At a mean relative abundance of 28.9%, Cyanobiaceae remained the dominant Cyanobacteria subtaxa with similar structural observations across all samples. Notably, the highly potent pathogenic family Vibrionaceae and Flavobacteriaceae were observed to have occasional spikes in abundance in the southern FF sites during February and in the RS sites during June, respectively. Among eastern AFF sites, temporal shift in bacterial community compositions were uniformly observed in February, characterized by the reduction in Cyanobacteria subtaxa (from an average of 34.8% across all other months to 2.7% in February).

### Aquaculture inactivity directly reduced total bacterial community diversity

**Figure 4:**
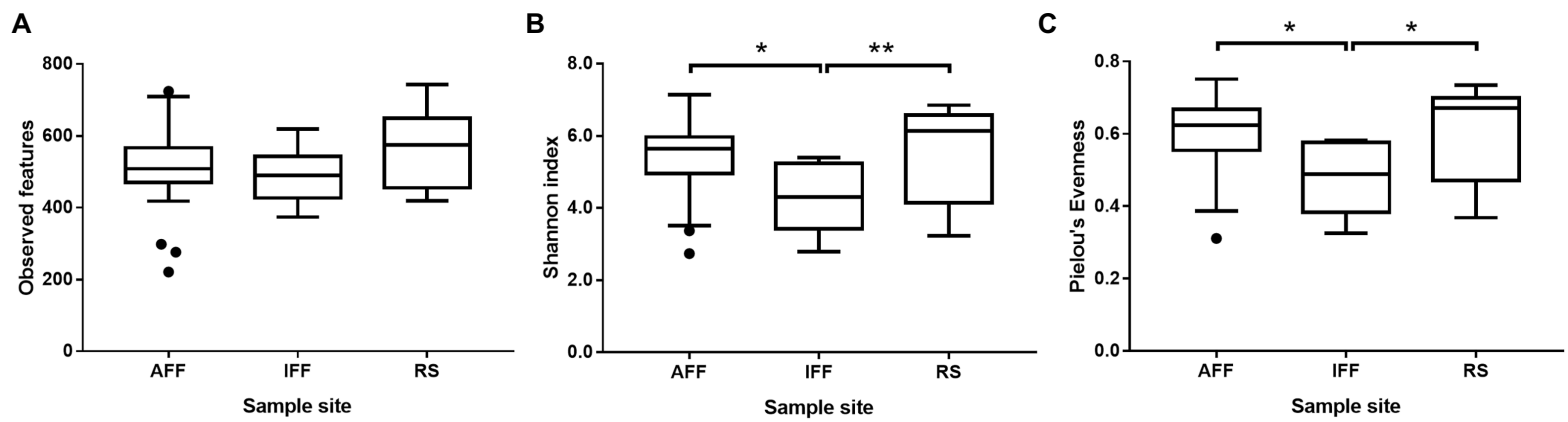
Variation in alpha diversity indices including **(A)** observed features, **(B)** Shannon index, and **(C)** Pielou’s evenness, among bacterial communities in sampled Hong Kong coastal waters. Asterisks indicate statistically significant difference (Kruskal-Wallis test, *p* < 0.05). AFF, active fish farm; IFF, inactive fish farm; RS, reference site. Asterisks indicate statistically significant difference according to corresponding statistical tests mentioned in figure i.e., Mann Whitney U-test or Kruskal-Wallis test. * indicates *p* < 0.05 and ** indicates *p* < 0.01.

Alpha diversity indices including observed features, Shannon index and Pielou’s evenness were used to evaluate bacterial diversity of Hong Kong coastal waters and were summarized in Figure 4 (see Supplementary Table 2 for detailed information). Overall results showed that AFF had significantly higher Shannon diversity index and Pielou’s evenness when compared to IFF but was indifferent to RS. This suggested that observed dominant compositional shift in IFF resulted in reduced community diversity beyond simple species richness, while AFF-adapted community may more closely resemble the status of open waters.

**Figure 5:**
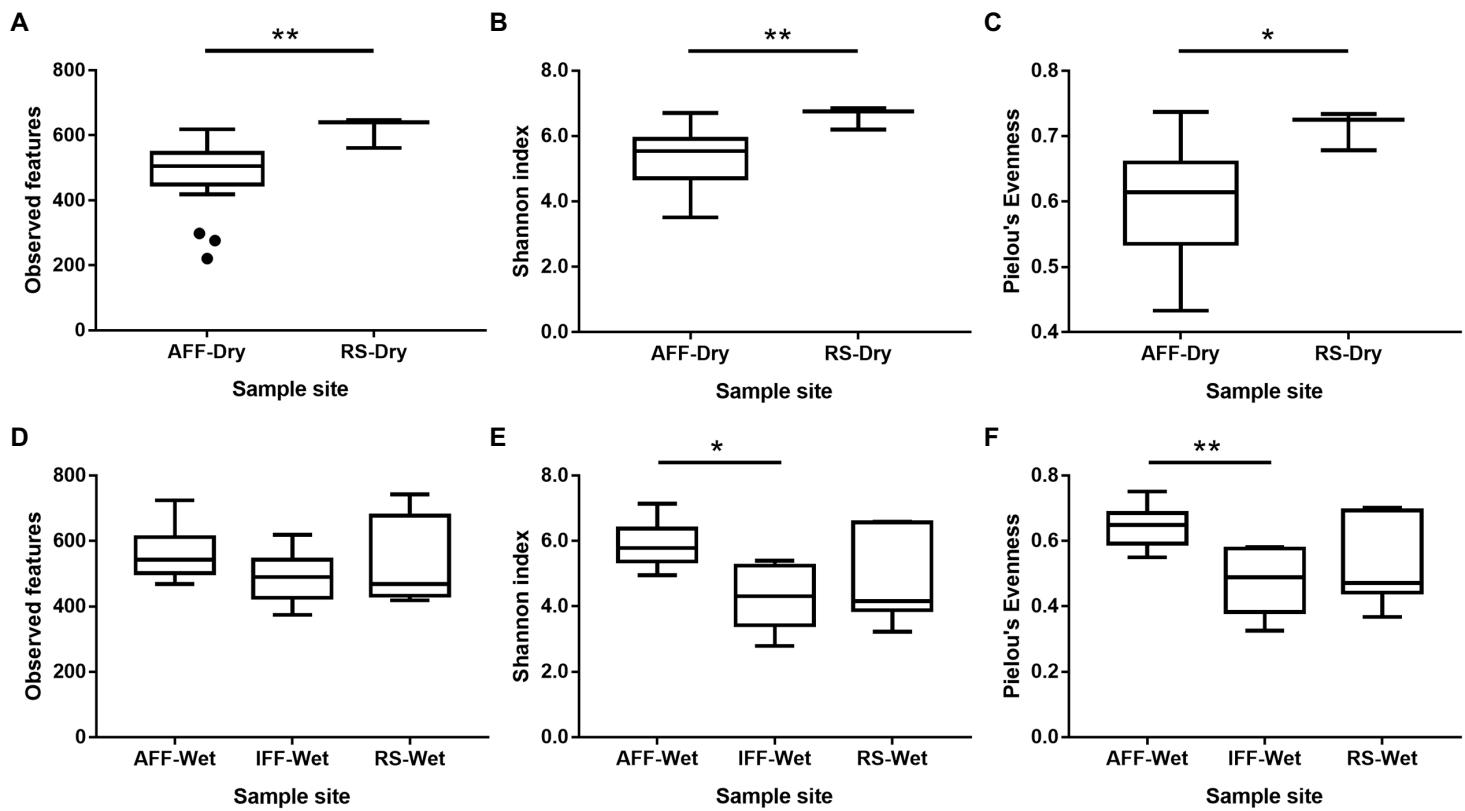
Variation in alpha diversity indices among bacterial communities in sampled Hong Kong coastal waters during **(A–C)** dry and **(D–F)** wet seasons. Asterisks indicate statistically significant difference (**A–C**, Mann–Whitney U test; **D–F**, Kruskal-Wallis test; *p* < 0.05). AFF, active fish farm; IFF, inactive fish farm; RS, reference site. Asterisks indicate statistically significant difference according to corresponding statistical tests mentioned in figure i.e., Mann Whitney U-test or Kruskal-Wallis test. * indicates *p* < 0.05 and ** indicates *p* < 0.01.

As IFFs were mainly identified to occur during the wet season of June, a potential seasonal rainfall impact on bacterial community composition and diversity was considered and further investigated. In the following analysis, samples were first classified based on sampling season and associated rainfall patterns, where dry season consisted of samples from November to February and wet season consisted of samples from June to August. In the dry season where the influence of rainfall dilution and nearby run-off is relatively low, AFF showed a consistently lower bacterial alpha diversity compared to RS. This may represent a potential loss in ecological niches and service contributors due to fish farming activities, as shown in Figures 5A–C (Mann–Whitney U test, *p* < 0.05). In the wet season, no similar patterns in alpha diversity were observed as AFF were not significantly different from RS. However, IFF observed in this season had significantly lower Shannon diversity and Pielou’s evenness compared to AFF sites (Figures 5D-F; Kruskal-Wallis test, *p* < 0.05), indicating a potentially slow community recovery after reduced human influence and nutrient load from fish farming activities. Between the two seasons, AFF samples did not display significant difference in alpha diversity, while RS samples were greatly affected by seasonal difference and dilution effects, resulting in lower alpha diversity (Mann–Whitney U test, *p* < 0.05).

**Figure 6:**
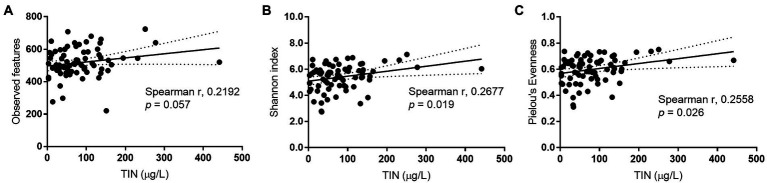
Spearman’s correlation between bacterial alpha diversity indices **(A)** observed features, **(B)** Shannon index, **(C)** Pielou’s Evenness, and total inorganic nitrogen concentrations in sampled Hong Kong coastal waters. Significance is recognized when *p* < 0.05.

To investigate the reduction in alpha diversity observed in IFFs, Spearman’s correlation analysis was conducted. Results showed that among all recorded physicochemical parameters, only TIN concentration which distinguished IFFs from AFFs could be an explanatory factor for increasing bacterial alpha diversity. Factors such as temperature, pH, and chlorophyll-a concentration showed no significant correlation (*p* > 0.05). A significant positive correlation was identified between TIN and Shannon diversity and Pielou’s evenness (*p* < 0.05), as shown in Figures 6A-C. Low TIN conditions has the potential to be a limiting factor for alpha diversity which may have an impact on bacterial ecological services and community stability.

### Aquaculture activity as a key co-influencing factor on bacterial beta diversity and differentially abundant taxa

**Figure 7:**
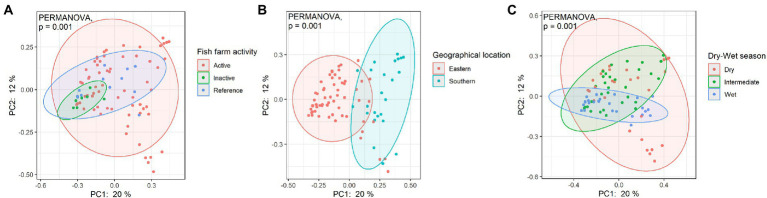
Bray-Curtis PCoA of bacterial communities among different Hong Kong coastal waters as grouped according to **(A)** fish farming activities, **(B)** geographic location of sample site, and **(C)** sampling season. Significant difference was tested with PERMANOVA, *p* < 0.05.

PCoA was used to further visualize and explore the dissimilarities between sampled bacterial community structures and beta diversity. When samples were grouped based on fish farming activities, geographic location, or dry-wet season, PCoA results showed that all site groups were each dissimilar, as shown in Figure 7. A significant difference between categorical groups was identified (PERMANOVA, *p* = 0.001), indicating the spatiotemporal influence and human impact on coastal bacterial communities. An ADONIS test was conducted to test the individual main effects on bacterial beta diversity. Results showed that spatial variation in geographic location, dry/wet seasonal difference, and fish farming activities explained 12.1, 7.9, and 7.5% of the variation, respectively.

**Figure 8:**
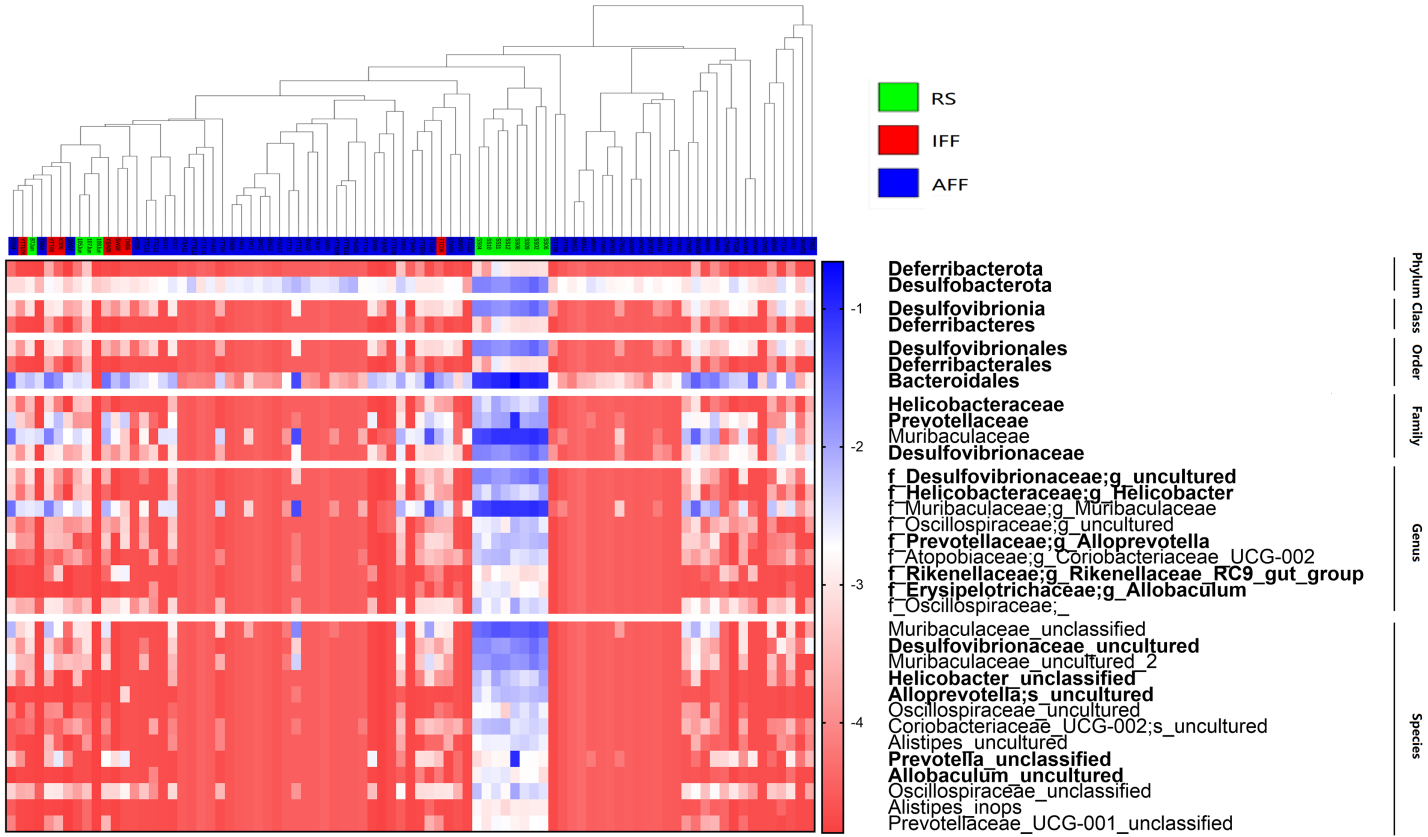
Relative abundance heatmap showing the bacterial taxa which were significantly differentially abundant between FF (IFF and AFF) and RS groups as revealed by ANCOM W-values after false discovery rate correction at each taxonomic rank. Color gradient (red to blue) of heatmap plot represents log transformed relative abundance of each taxon (rows) within each sample (columns). Bacterial taxa are bolded if it is classified as a contributor to nitrogen cycle-related KEGG pathways. The samples in the dendrogram was clustered and color-coded to represent FF and RS site groups. AFF, active fish farms; IFF, inactive fish farms; RS, open water reference sites.

The hierarchal clustering of sampled communities based on fish farm activities was followed by an ANCOM to reveal the differentially abundant bacterial taxa with considerations to community compositionality, as shown in Figure 8. The subtaxa of phyla Deferribacterota and Desulfobacterota and order Bacteroidales were notable differentially abundant taxa. These taxa are identified contributors to denitrification and dissimilatory nitrate reduction pathways within the nitrogen cycle, with enriched relative abundances in the RS. From our results, the order Bacteroidales can occupy up to 22% of the bacterial community relative abundance in RS, while Deferribacterota and Desulfobacterota typically accounted for 1–3% of relative abundance in RS. These rare taxa of low abundance and high diversity represented a notable proportion of the community. However, no significant increase in nitrogen cycle-related functional abundance which can be associated with the currently observed differentially abundant taxa of Bacteroidales, Deferribacterota and Desulfobacterota. Whether there is potential enrichment of specific bacterial taxa by fish farming activities, however, was not detected as all highlighted taxa in Figure 8 had reduced relative abundances in FFs.

### Nitrogen cycle-related bacterial functions determined by bacterial selection in fish farming waters

**Figure 9:**
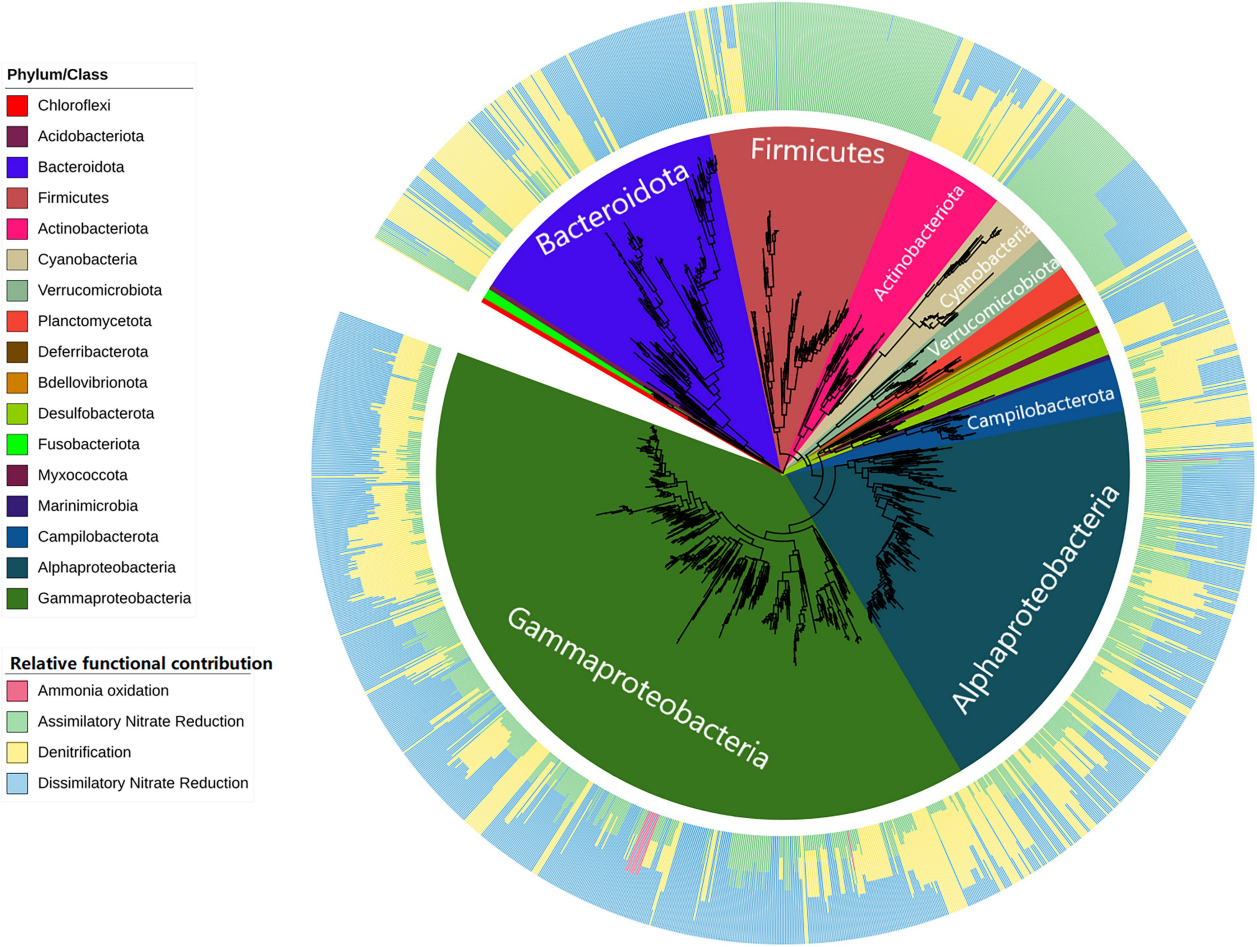
Phylogenetic tree of nitrogen cycle-related bacteria predicted by PICRUSt2 in Hong Kong coastal waters. The inner circle plot indicates corresponding phylum, or class taxonomy for phylum Proteobacteria, of the phylogenetic tree. The outer circle plot indicates the proportion of nitrogen cycle-related functional abundance assigned to individual nitrogen cycling pathways within the taxon.

The PICRUSt2 tool was used to infer the functional potential of coastal bacterial communities. In particular, the bacterial ASVs contributing to aquatic nitrogen cycle-related pathways as predicted by PICRUSt2 were selected for further investigation due to their implications for aquatic and fish farming-related environments. Overall, a total of 1,695 ASVs were found to contribute to nitrogen cycle-related pathways. This translated into a diverse range of bacterial taxa covering 663 species among 424 genera. We found that for the majority of taxa excluding Firmicutes and Cyanobacteria, they tended to also harbor genes associated with denitrification if they already encode those for dissimilatory nitrate reduction, as shown in Figure 9. Observed Firmicutes subtaxa are likely specialists of assimilatory nitrate reduction. Additionally, Nitrosococcaceae was the only family of bacteria inferred to contribute to ammonia oxidation. There were no anammox-specific genes detected.

**Figure 10:**
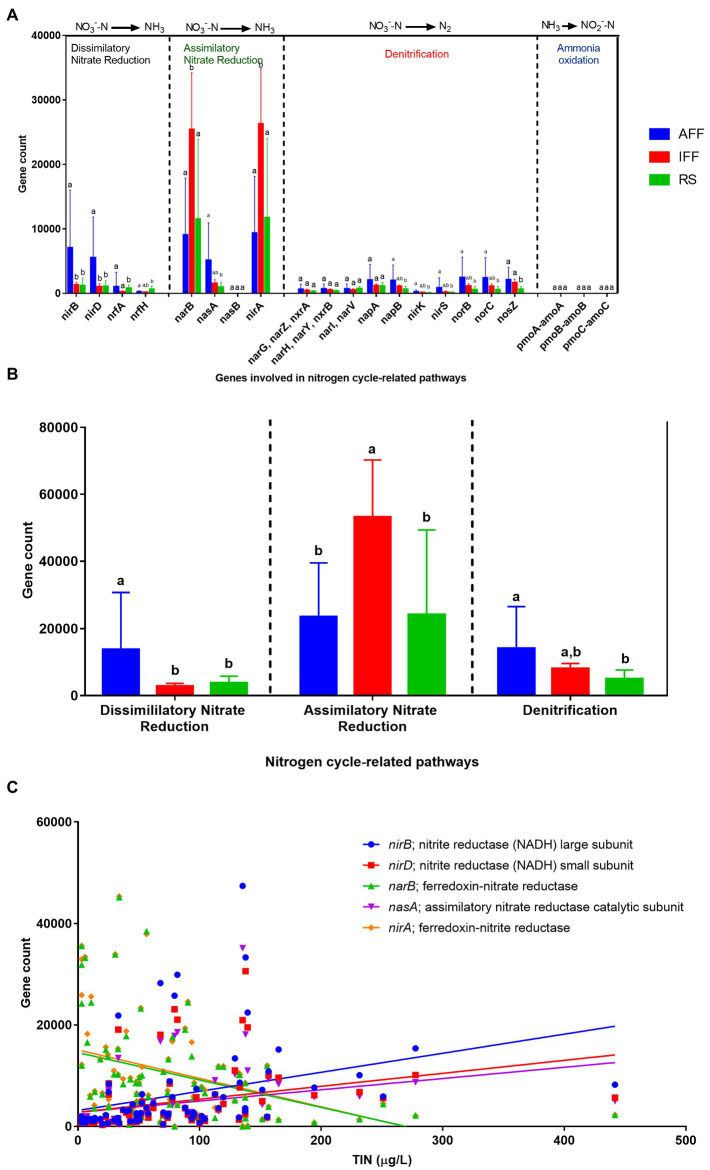
Summary of the mean abundance of **(A)** nitrogen cycle-related pathways and **(B)** the contributing genes, as well as **(C)** Spearman’s correlation between major nitrogen cycle-related genes and total inorganic nitrogen concentration in Hong Kong coastal bacterial communities. Different letters indicate statistically significant differences (Kruskal-Wallis test, *p* < 0.05). AFF, active fish farm; IFF, inactive fish farm; RS, reference site.

The specific genes harbored by coastal bacterial communities contributing to nitrogen cycle-related pathways were summarized in Figure 10. The processes shown were dissimilatory and assimilatory nitrate reduction, denitrification, and ammonia oxidation. For dissimilatory nitrate reduction, nitrite reductase genes *nirB* followed by *nirD* were the most abundant associated genes recorded with a significantly higher abundance in AFF sites. Both *nirB* and *nirD* were more than five times more abundant in AFF sites than IFF and RS. For assimilatory nitrate reduction, the *narB* and *nirA* genes were the most dominantly abundant genes largely observed in IFF sites; their mean gene counts were, on average, more than twice as common compared to AFF and RS. A total of ten genes associated with the denitrification pathway were identified. The majority of these genes were found to be distributed relatively equally at low abundance among coastal waters. However, when their total pathway abundance was considered, a significantly higher total abundance was revealed for AFF sites, which may indicate a higher rate of process. Lastly, the *pmoABC-amoABC* genes were found to be rare among Hong Kong coastal bacterial communities with a uniformly much lower and spontaneous abundance across sites, which is to be expected for ammonia oxidation pathways contributed by very limited and specialized taxa. Their prevalence was too low and omitted in the subsequent analysis. Spearman’s correlation analysis between the top five abundant nitrogen cycle-related genes and TIN concentration revealed significant correlations that reinforced the results from Figure 10B. The abundance of genes *nirB*, *nirD* and *nasA* were significantly positively correlated with TIN concentration (*r* > 0.55, *p* < 0.01), while the opposite was identified for genes *narB* and *nirA* (*r* < −0.45, *p* < 0.01).

## Discussion

In this study, the dedicated aquaculture zones in Hong Kong provided a useful model to understand microbial community dynamics and functional contributions affected by aquaculture input and practice. Both elevated and continuous nitrogen input from active aquaculture as well as fallow periods were drivers of change behind planktonic microbial compositions and their nitrogen cycling functional potentials. In addition to the results, the study provided new insights to the roles played by dominant and rare bacterial taxa, and identified previously underexplored contributors within the nitrogen cycling bacterial community in the context of aquaculture nitrogen load, these ideas are further discussed below.

### The influence of enriched nitrogen load and aquaculture activities on dominant and rare bacterial compositions

Excessive nitrogen loading is a known direct interference with the original nitrogen cycle in any water system not limited to aquaculture areas. There has been focused attention devoted to understanding its implications for nitrogen balance and footprint, mainly revolving around wastewater treatment facility sources and management strategies (Yu et al., 2019). In aquaculture, nitrogen application and management are also key components to increase output productivity and utilization efficiency (Luo et al., 2018), as well as important challenges for protecting water quality. In this regard, the current trend of intensifying aquaculture and water nitrogen load has seen less exploration from a microbial perspective.

From the current study, we first identified a generally higher TIN concentration in southern fish farms with likely elevated nitrogen input and water nitrogen load. The dominant taxa in the total bacterial community are often considered the most important in community stability and maintenance (Jiao et al., 2019). On this foundation, we observed that Proteobacteria classes, namely Alpha- and Gammaproteobacteria, were enriched during active fish farming periods across all fish farms, and it is reasonable to infer inorganic nitrogen in seawater as a potential major contributing factor (Wang et al., 2023). Using TIN as an intuitive gradient, we further observed significantly reduced alpha diversity in inactive fish farms compared to its active counterparts, both during the wet season and throughout the year. However, it could be more appropriate to attribute this observation to the removal of bacteria associated with finfish hosts during the temporary fallow period, than to the potential loss of diversity with reduced TIN concentrations. From another perspective, the short fallow period in fish farm waters could represent a designated recovery period. From our results, bacterial communities in temporarily fallowed fish farms were characterized by increase in Cyanobacteria relative abundance. It is known that Cyanobacteria can adapt to and develop in nutrient-deficient environments, but they also are potential bloom species (Kim et al., 2004). More long-term evidence will be required for an informed suggestion on the feasibility and recovery of coastal sites under direct aquaculture impact (Verhoeven et al., 2018).

In addition to dominantly abundant taxa, rare taxa of high diversity and low abundance were also suggested to be important in many ecosystem functions and promote community stability and multifunctionality as the more responsive subcommunity to environmental disturbance (Mo et al., 2018). The importance of rare taxa in ecosystem functionality has been recently reported in the likes of soil–plant systems (Xiong et al., 2021), grassland (Soliveres et al., 2016), and wetland ecosystems (Zhang N. et al., 2022). Subsequently, ANCOM analysis revealed several rare taxa to be differentially abundant in relation to aquaculture activity, such as the subtaxa of Desulfobacterota; they have significantly higher relative abundances in open water reference sites. Notably, Desulfobacterota is a recently enacted phylum that encompasses most sulfate-reducing bacteria (Waite et al., 2020). This may indicate the taxon’s potentially limited adaptability to human-manipulated niches, such as those under aquaculture influence, and requires further empirical exploration.

### Dominant cyanobacterial contributions to predicted nitrogen-cycling services following reduction in nitrogen load

Microbial nitrogen cycling is the most well studied bacteria-dominated biogeochemical cycle process across a wide range of mostly terrestrial habitats. In this aquatic ecosystem, our study demonstrated that fish farming activities were significant sources of inorganic nitrogen availability, which in turn has an impact on predicted functional contributions provided by the nitrogen-cycling microbial community. In terms of functional abundance across all studied coastal waters, we report cyanobacteria *Synechococcus* sp. CC9902 to be by far the most abundant taxa annotated to predicted nitrogen cycling, with relative abundance reaching up to 57% among total bacterial communities in IFFs with reduced nitrogen load. A higher abundance of sequences annotated with nitrogen cycling function is typically assumed to be indicative of faster nutrient cycling rates (Nelson et al., 2016). This however requires careful examination as Rocca et al. (2015) showed that there was often little correlation between general gene abundance and the rate of actual functional process it is annotated to. For instance, Cyanobacteria are photosynthetic autotrophs well capable of occurring in nutrient-rich and oligotrophic conditions (Wang et al., 2021). Despite their functional annotation, Cyanobacteria are not limited to using nitrate; other nitrogen sources utilized by Cyanobacteria include ammonium, urea, and dinitrogen. It is known that ammonium is the preferred nitrogen source, and as a result, genes encoding enzymes that assimilate alternative nitrogen sources are known to be repressed during the presence of ammonium. For example, genes *nirA* and *narB* in this study corresponding to nitrite reductase and nitrate reductase are both only expressed at high levels in the absence of ammonium (Herrero et al., 2001). Hence, the relative contribution by Cyanobacteria may not be rigorously represented. Nonetheless, this study demonstrated that in aquaculture and human manipulated systems, inference of functional potential is a helpful tool for investigating the roles of microbial communities in biogeochemical cycles, but its interpretation should be handled with care. Future studies should incorporate metatranscriptomics to provide further evidence of bacterial compositional and functional expression and adaptations.

### Fish farm selection of Rhodobacteraceae involved in marine nitrogen cycle and potential indication of phytoplankton bloom

The following most abundant group of nitrogen cycling bacteria were Alpha- and Gammaproteobacteria. Compared to Cyanobacteria, their taxonomic and nitrogen cycle-related functional composition were both much more diverse, as shown to cover more than half of the contributing phylogenetic diversity. Among Alphaproteobacteria, the family Rhodobacteraceae contributed 70% of its predicted functional abundance, while no such dominant family can be identified for Gammaproteobacteria. Rhodobacteraceae are known to be aerobic photoheterotrophs and chemoheterotrophs involved in sulfur and carbon biogeochemical cycling (Pujalte et al., 2014). Their prevalence in contribution toward sulfur and nitrogen cycle and metabolism-related functionality has been recently reported along the Southern California Bight coastline (Li et al., 2018). In urbanized estuarine areas, patterns in bacterial composition of Rhodobacteraceae, Flavobacteraceae, *Synechococcus* and more were reportedly influenced by dynamic nutrient input (Jeffries et al., 2016). It is also remarkable that Rhodobacteraceae can be closely linked to phytoplankton. High abundances of Rhodobacteraceae have reportedly co-occurred with blooms of phytoplankton (Eilers et al., 2001) and rapidly responded to the growth and decay of different phytoplankton (Grossart et al., 2005). This may suggest a joint relationship between aquaculture water nitrogen load, bacterial communities, and potential bloom risks reflected by Rhodobacteraceae abundance.

In terms of nitrogen-related functional pathways, dissimilatory nitrate reduction to ammonium (DNRA) was the second most abundant pathway and the function was predicted in majority of the Proteobacteria. Although the contributing genes have been frequently detected in bacteria from various systems (Zhang and Lv, 2021), the key taxonomic contributors to DNRA have not been thoroughly explored compared to other major nitrogen cycling processes such as denitrification (Decleyre et al., 2015). From our results, we report the family Nitrincolaceae (under Gammaproteobacteria) and Rhodobacteraceae (under Alphaproteobacteria) to be the most abundant contributing family predicted for nitrite reductase *nirB* and *nirD* gene abundance in active fish farm waters. Additionally, Nitrosococcaceae was the only family of Gammaproteobacteria inferred to contribute to ammonia oxidation. It is known that the ability to perform ammonia oxidation and nitrification is restricted to several microbial taxa (Soliman and Eldyasti, 2018). Despite its rare occurrence, notably, the abundance of Nitrosococcaceae was found to be concentrated from September to December during the active period of fish farm sites. These observations may serve as the first steps to elucidate potential selection imposed under a coastal aquaculture setting.

## Conclusion

Coastal planktonic microbial communities in aquaculture sites showed shifts in dominant taxonomic profiles including Proteobacteria, Cyanobacteria, and Bacteroidales with ongoing active aquaculture, which has knock-on impact on their alpha diversity and predicted functional contributions in nitrogen cycle-related pathways. This variation in predicted functional abundance can be explained by the regional difference in aquaculture practice defined by inorganic nitrogen load levels. We identified a dominant increase in predicted genes contributing to assimilatory nitrate reduction pathway in fallowed fish farms, indicating total bacterial and further nitrogen cycling subcommunity could be sensitive and rapidly selected by changes such as a short hiatus in coastal aquaculture practices. Whether such hiatus in aquaculture are effective recovery strategies for coastal water quality, however, still requires further studies. This study provided new insights on the microbial response and interactions with aquaculture activities that has wide coastal implications.

## Data availability statement

The datasets presented in this study can be found in online repositories. The names of the repository/repositories and accession number(s) can be found in the article and below: National Center for Biotechnology Information (NCBI; https://www.ncbi.nlm.nih.gov); accession number PRJNA908141.

## Author contributions

LL, ZX, SL, and WL performed data curation, methodology, and investigation. LL and JC performed investigation, formal analysis, and writing of the manuscript. J-WQ provided resources and commented on the writing of the manuscript. HL and P-YQ provided resources, supervision, and revised the manuscript. JC also performed conceptualization, resources, supervision, and funding acquisition of the work. All authors contributed to the article and approved the submitted version.

## Funding

This research was supported by Southern Marine Science and Engineering Guangdong Laboratory (Guangzhou) (SMSEGL20SC01), and the General Research Fund by the Research Grants Council of the Hong Kong Special Administrative Region, China (Project No. 16101821).

## Conflict of interest

The authors declare that the research was conducted in the absence of any commercial or financial relationships that could be construed as a potential conflict of interest.

## Publisher’s note

All claims expressed in this article are solely those of the authors and do not necessarily represent those of their affiliated organizations, or those of the publisher, the editors and the reviewers. Any product that may be evaluated in this article, or claim that may be made by its manufacturer, is not guaranteed or endorsed by the publisher.

## Supplementary material

The Supplementary material for this article can be found online at: https://www.frontiersin.org/articles/10.3389/fmicb.2022.1062029/full#supplementary-material

## Footnotes

^1^ https://cd.epic.epd.gov.hk/EPICRIVER/marine/
