# Supplementary material for: How elevated nitrogen load affects bacterial community structure and nitrogen cycling services in coastal water
Source: Front Microbiol. 2022 Dec 22;13:1062029. doi: 10.3389/fmicb.2022.1062029 (PMC9815024; doi:10.3389/fmicb.2022.1062029)
Supplement: Supplementary file 1 [file Data_Sheet_1.PDF]

How elevated nitrogen load affects bacterial community structure and nitrogen cycling services in coastal water

Linus Shing Him Lo[1, 2], Zhimeng Xu[1, 2], Sangwook Scott Lee [1, 2], Wing Keung Lau [1], Jian-Wen Qiu [2, 3], Hongbin Liu[1,2]*, Pei-Yuan Qian[1, 2]*, Jinping Cheng [1, 2, 4]*

1 Department of Ocean Science, The Hong Kong University of Science and Technology, Hong Kong SAR, China

2 The Southern Marine Science and Engineering Guangdong Laboratory (Guangzhou), Guangzhou, China

3 Department of Biology, Hong Kong Baptist University, Hong Kong SAR, China

4 Department of Science and Environmental Studies, The Education University of Hong Kong, New Territories, Hong Kong SAR, China

Supplementary Table 1. Summary of environmental parameters of collected Hong Kong fish farm water samples.

Sampling date	Sample site	Station name	Location	Season	Temperature (°C)	pH	Dissolved oxygen (mg/L)	Total inorganic nitrogen (μg/L)	Chlorophyll-a (μg/L)
2020/9/16	E4	SW	Eastern	Intermediate	29.273	8.040	7.640	13.360	3.610
2020/9/16	E5	TM	Eastern	Intermediate	29.151	7.930	7.840	29.570	0.990
2020/9/16	E2	YTT	Eastern	Intermediate	29.296	8.270	8.350	61.960	3.880
2020/9/23	S1	LTW	Southern	Intermediate	28.977	7.990	6.080	277.530	4.280
2020/9/23	S3	MW	Southern	Intermediate	29.254	8.020	5.810	441.630	1.520
2020/9/23	S2	SKW	Southern	Intermediate	28.899	8.000	6.140	253.790	6.300
2020/9/29	E1	YSA	Eastern	Intermediate	28.635	8.170	7.370	47.610	6.970
2020/9/29	E3	YTTE	Eastern	Intermediate	27.849	7.800	6.920	73.720	5.860
2020/10/5	E4	SW	Eastern	Intermediate	29.323	8.180	7.420	50.280	2.600
2020/10/5	E5	TM	Eastern	Intermediate	29.268	8.220	7.350	50.550	2.950
2020/10/5	E2	YTT	Eastern	Intermediate	29.777	8.390	2.180	101.590	11.690
2020/10/6	S1	LTW	Southern	Intermediate	28.819	8.140	6.810	129.060	4.540
2020/10/6	S3	MW	Southern	Intermediate	28.942	8.110	6.130	194.340	2.150
2020/10/6	S2	SKW	Southern	Intermediate	28.496	8.100	6.650	137.460	6.300
2020/10/8	E6	KS	Eastern	Intermediate	28.337	8.180	6.580	51.570	4.480
2020/10/8	E7	TTC	Eastern	Intermediate	27.901	8.180	6.540	88.080	7.350
2020/10/14	E1	YSA	Eastern	Intermediate	28.032	8.050	4.400	155.570	5.740
2020/10/14	E3	YTTE	Eastern	Intermediate	27.383	8.120	6.330	95.560	3.410
2020/11/4	E4	SW	Eastern	Dry	24.972	8.130	7.590	13.810	3.120

2020/11/4	E5	TM	Eastern	Dry	24.902	8.130	7.500	43.040	3.210
2020/11/5	S1	LTW	Southern	Dry	24.432	7.990	7.020	156.690	9.250
2020/11/10	E3	YTTE	Eastern	Dry	24.487	8.120	7.720	32.890	4.770
2020/11/16	E7	TTC	Eastern	Dry	23.034	8.150	8.830	N/A	N/A
2020/11/17	E1	YSA	Eastern	Dry	24.385	8.020	5.800	18.350	5.810
2020/11/17	E2	YTT	Eastern	Dry	24.791	8.210	8.310	57.800	7.280
2020/11/18	S3	MW	Southern	Dry	23.910	8.030	6.730	231.530	2.110
2020/11/18	S2	SKW	Southern	Dry	23.494	8.010	7.180	164.910	3.960
2020/12/1	E4	SW	Eastern	Dry	22.360	8.060	7.290	132.460	2.250
2020/12/1	E1	YSA	Eastern	Dry	24.376	8.000	5.450	24.590	6.200
2020/12/1	E3	YTTE	Eastern	Dry	23.740	7.800	4.220	123.330	2.990
2020/12/21	E5	TM	Eastern	Dry	18.325	8.080	7.730	104.320	1.160
2020/12/21	E2	YTT	Eastern	Dry	19.451	7.960	7.130	94.930	2.750
2020/12/22	S1	LTW	Southern	Dry	19.188	7.980	7.620	67.740	0.780
2020/12/22	S3	MW	Southern	Dry	19.337	7.990	7.220	151.540	0.760
2020/12/22	S2	SKW	Southern	Dry	19.180	7.930	7.590	48.190	1.070
2020/12/23	E6	KS	Eastern	Dry	18.454	7.980	8.030	90.400	1.260
2021/2/2	S1	LTW	Southern	Dry	18.108	8.220	8.770	81.730	1.130
2021/2/2	S3	MW	Southern	Dry	18.293	8.210	8.410	137.860	2.210
2021/2/2	S2	SKW	Southern	Dry	18.129	8.210	8.820	79.430	1.050
2021/2/3	E5	TM	Eastern	Dry	17.650	8.230	9.130	135.340	0.510
2021/2/3	E2	YTT	Eastern	Dry	19.439	8.230	8.330	139.460	1.090
2021/2/4	E6	KS	Eastern	Dry	17.903	8.180	8.670	33.070	0.480

2021/2/19	E7	TTC	Eastern	Dry	19.815	8.160	7.580	25.300	0.830
2021/3/1	E7	TTC	Eastern	Intermediate	20.238	8.200	8.140	74.300	1.110
2021/3/23	E1	YSA	Eastern	Intermediate	20.889	8.130	6.630	115.500	5.420
2021/4/7	E4	SW	Eastern	Intermediate	23.274	7.970	6.180	30.580	6.250
2021/4/7	E3	YTTE	Eastern	Intermediate	24.246	7.840	5.630	70.170	5.970
2021/4/8	E6	KS	Eastern	Intermediate	23.450	7.900	6.780	33.860	2.090
2021/4/12	E5	TM	Eastern	Intermediate	23.533	8.000	6.990	56.410	5.690
2021/4/12	E2	YTT	Eastern	Intermediate	23.923	7.810	6.700	77.570	10.510
2021/4/14	S1	LTW	Southern	Intermediate	24.366	7.980	6.700	133.030	1.130
2021/4/14	S3	MW	Southern	Intermediate	24.962	7.970	7.090	250.930	2.530
2021/4/14	S2	SKW	Southern	Intermediate	24.207	7.990	6.770	119.520	1.050
2021/5/3	E6	KS	Eastern	Intermediate	25.030	7.930	6.960	3.000	0.990
2021/5/13	S2	SKW	Southern	Intermediate	27.147	8.060	7.830	52.680	1.430
2021/5/27	E1	YSA	Eastern	Intermediate	29.591	7.960	6.700	3.000	1.530
2021/6/2	E7	TTC	Eastern	Wet	27.620	8.020	7.700	3.000	7.740
2021/6/3	E4	SW	Eastern	Wet	27.678	8.000	7.640	10.780	1.010
2021/6/3	E3	YTTE	Eastern	Wet	28.095	7.950	7.860	3.000	2.440
2021/6/8	E5	TM	Eastern	Wet	26.245	7.960	6.700	7.610	0.450
2021/6/8	E2	YTT	Eastern	Wet	28.570	8.050	8.480	5.840	1.460
2021/6/10	S1	LTW	Southern	Wet	26.970	8.060	7.970	42.220	9.180
2021/6/10	S3	MW	Southern	Wet	27.385	8.100	7.830	97.300	3.700
2021/7/5	E7	TTC	Eastern	Wet	29.207	8.230	8.690	75.330	3.010
2021/8/4	S1	LTW	Southern	Wet	28.872	8.230	9.690	44.970	1.940

2021/8/4	S3	MW	Southern	Wet	28.790	8.270	10.400	112.630	7.560
2021/8/4	S2	SKW	Southern	Wet	28.922	8.240	9.510	52.470	1.760
2021/8/10	E5	TM	Eastern	Wet	25.697	7.910	6.290	48.260	0.910
2021/8/10	E2	YTT	Eastern	Wet	28.433	7.980	6.210	25.150	7.060
2021/8/16	E6	KS	Eastern	Wet	26.247	7.910	6.860	22.850	0.650
2021/8/17	E4	SW	Eastern	Wet	27.442	8.020	6.610	93.500	0.650
2021/8/17	E3	YTTE	Eastern	Wet	28.418	8.010	7.030	37.140	1.950
2021/8/24	E1	YSA	Eastern	Wet	27.894	7.900	4.450	39.690	1.920

Supplementary Table 2. Alpha diversity indices of total bacteria community in sampled Hong Kong coastal waters.

Sample site-month	Feature Count	Observed features	Shannon Index	Pielou's evenness	Sample site-month	Feature Count	Observed features	Shannon Index	Pielou's evenness
**Active fish farm sites**					**Inactive fish farm sites**				
E1-03	52266	534	5.601	0.618	E1-05	48109	486	4.368	0.489
E1-08	57466	512	5.539	0.615	E2-06	52437	490	3.423	0.383
E1-09	30739	456	5.888	0.667	E3-06	50937	374	2.789	0.326
E1-10	29952	501	5.159	0.575	E4-06	56737	427	4.307	0.493
E1-11	32653	514	4.664	0.518	E5-06	41312	543	5.240	0.577
E1-12	36998	419	4.937	0.567	E6-05	54352	508	3.868	0.430
E2-02	47988	298	4.021	0.489	E7-06	47302	619	5.396	0.582
E2-04	44839	487	4.965	0.556					
E2-08	50267	469	5.193	0.585	**Reference sites**				
E2-09	34128	490	5.866	0.656	R1-02	22637	561	6.195	0.678
E2-10	28862	471	5.848	0.659	R1-04	17989	549	6.075	0.668
E2-11	29123	478	5.951	0.669	R1-06	30644	656	6.568	0.702
E2-12	29782	533	6.391	0.706	R1-08	33952	743	6.580	0.690
E3-04	50008	580	4.459	0.486	R1-09	26331	650	6.299	0.674
E3-08	45184	475	5.782	0.650	R1-10	24867	587	5.937	0.645

E3-09	32350	463	6.234	0.704
E3-10	30880	459	5.857	0.662
E3-11	25943	444	4.815	0.548
E3-12	34643	479	4.659	0.523
E4-04	62400	439	3.667	0.418
E4-08	53308	503	5.643	0.629
E4-09	25971	568	6.747	0.737
E4-10	30109	582	6.002	0.653
E4-11	26531	484	5.771	0.647
E4-12	32841	523	5.547	0.614
E5-02	48415	276	3.512	0.433
E5-04	58679	424	3.369	0.386
E5-08	31781	639	5.366	0.576
E5-09	36497	499	5.756	0.642
E5-10	29475	546	5.649	0.621
E5-11	31859	454	5.542	0.628
E5-12	30565	471	5.940	0.669
E6-02	40343	597	5.763	0.625
E6-04	62900	451	2.738	0.311
E6-08	41718	512	4.948	0.550
E6-10	45010	504	4.340	0.483
E6-12	46956	618	4.488	0.484
E7-02	49609	453	5.684	0.644
R1-11	19896	640	6.755	0.725
R1-12	26948	646	6.849	0.734
R2-06	45857	441	4.107	0.467
R3-06	45790	496	4.224	0.472
R4-06	59004	419	4.102	0.471
R5-06	55272	438	3.232	0.368

E7-03	46533	488	5.103	0.571
E7-07	51745	584	6.140	0.668
E7-10	43602	475	4.718	0.531
E7-11	44115	581	5.103	0.556
S1-02	46293	418	4.760	0.547
S1-04	48843	630	5.361	0.576
S1-06	25455	566	6.404	0.700
S1-08	38350	562	6.442	0.705
S1-09	28393	600	6.637	0.719
S1-10	33505	641	6.153	0.660
S1-11	34335	601	5.884	0.637
S1-12	30585	532	5.453	0.602
S2-02	47828	221	3.841	0.493
S2-04	39867	519	4.750	0.527
S2-05	45082	500	5.370	0.599
S2-08	36950	709	6.368	0.672
S2-09	28678	474	5.763	0.648
S2-10	31514	453	5.404	0.612
S2-11	27530	546	6.435	0.708
S2-12	29606	505	5.838	0.650
S3-02	44134	511	5.241	0.583
S3-04	42126	662	5.660	0.604
S3-06	27977	724	7.135	0.751

S3-08	42033	543	5.895	0.649
S3-09	30195	681	6.884	0.731
S3-10	28933	521	6.038	0.669
S3-11	26056	546	6.646	0.731
S3-12	21659	545	6.700	0.737
